# Highly porous phosphate-based glasses for controlled delivery of antibacterial Cu ions prepared *via* sol–gel chemistry

**DOI:** 10.1039/d3ra02958a

**Published:** 2023-06-29

**Authors:** Farzad Foroutan, Benjamin A. Kyffin, Athanasios Nikolaou, Jorge Merino-Gutierrez, Isaac Abrahams, Nasima Kanwal, Jonathan C. Knowles, Andrew J. Smith, Glen J. Smales, Daniela Carta

**Affiliations:** a School of Chemistry and Chemical Engineering, University of Surrey Guildford UK d.carta@surrey.ac.uk; b School of Biosciences and Medicine, University of Surrey Guildford UK; c Department of Chemistry, Queen Mary University of London Mile End Road London E1 4NS UK; d Division of Biomaterials and Tissue Engineering, University College London London UK; e Department of Nanobiomedical Science & BK21 PLUS NBM Global Research Center for Regenerative Medicine, Dankook University Cheonan Republic of Korea; f UCL Eastman-Korea Dental Medicine Innovation Centre, Dankook University Cheonan Republic of Korea; g Diamond Light Source Ltd, Diamond House, Harwell Science and Innovation Campus Didcot, Oxfordshire OX11 0DE UK; h Bundesanstalt für Materialforschung und –prüfung (BAM) Berlin Germany

## Abstract

Mesoporous glasses are a promising class of bioresorbable biomaterials characterized by high surface area and extended porosity in the range of 2 to 50 nm. These peculiar properties make them ideal materials for the controlled release of therapeutic ions and molecules. Whilst mesoporous silicate-based glasses (MSG) have been widely investigated, much less work has been done on mesoporous phosphate-based glasses (MPG). In the present study, MPG in the P_2_O_5_–CaO–Na_2_O system, undoped and doped with 1, 3, and 5 mol% of Cu ions were synthesized *via* a combination of the sol–gel method and supramolecular templating. The non-ionic triblock copolymer Pluronic P123 was used as a templating agent. The porous structure was studied *via* a combination of Scanning Electron Microscopy (SEM), Small-Angle X-ray Scattering (SAXS), and N_2_ adsorption–desorption analysis at 77 K. The structure of the phosphate network was investigated *via* solid state ^31^P Magic Angle Spinning Nuclear Magnetic Resonance (^31^P MAS-NMR) and Fourier Transform Infrared (FTIR) spectroscopy. Degradation studies, performed in water *via* Inductively Coupled Plasma-Optical Emission Spectroscopy (ICP-OES), showed that phosphates, Ca^2+^, Na^+^ and Cu ions are released in a controlled manner over a 7 days period. The controlled release of Cu, proportional to the copper loading, imbues antibacterial properties to MPG. A significant statistical reduction of *Staphylococcus aureus* (*S. aureus*) and *Escherichia coli* (*E. coli*) bacterial viability was observed over a 3 days period. *E. coli* appeared to be more resistant than *S. aureus* to the antibacterial effect of copper. This study shows that copper doped MPG have great potential as bioresorbable materials for controlled delivery of antibacterial ions.

## Abstract 2

Mesoporous glasses are a promising class of bioresorbable biomaterials characterized by high surface area and extended porosity in the range of 2 to 50 nm.

## Graphical abstract

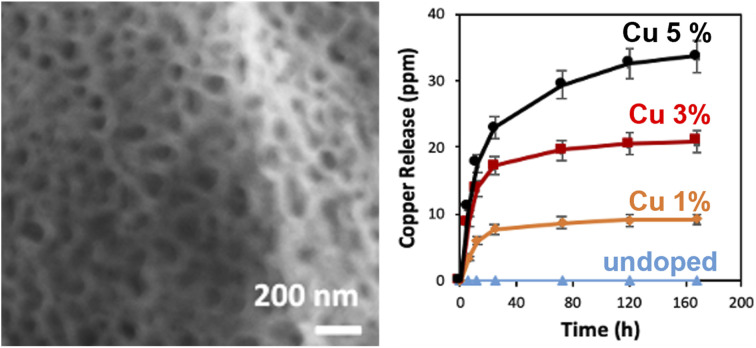

## 1. Introduction

Mesoporous glasses have been shown to have great potential as scaffolds for tissue engineering thanks to their peculiar properties of high specific surface area, tunable pore size, and the possibility of surface modification.^1^ In particular, the open porous structure, typical of mesoporous glasses, facilitates the absorption and delivery of therapeutic molecules and ions, making these materials excellent local drug delivery systems.^2^ In addition, mesoporous glasses have been shown to have enhanced biomedical properties compared to the non-porous analogous systems such as greater cytocompatibility and greater ability to induce the formation of hydroxy carbonated apatite, important properties in hard tissue engineering.^3^ The majority of mesoporous glasses used as biomaterials are silicate-based,^4^ and have been extensively studied as scaffolds for bone regeneration^5^ and more recently for wound healing.^6^ However, due to their low solubility, silicate-based glasses are mainly used to manufacture long-term implants which might be susceptible to failure and inflammatory reactions.^7–9^

Phosphate-based glasses (PG) represent a promising alternative to silicate-based systems. Being totally bioresorbable, they completely degrade over time in the physiological environment, being eventually entirely replaced by regenerated hard or soft tissues.^10,11^ PG also present advantages over bioresorbable polymers, whose degradation often results in crystalline fragments with heterogeneous chain-lengths that could lead to toxicity.^12^ Moreover, PG degradation products are already present in the body, reducing the risk of inflammatory reactions. PG are therefore the ideal materials for temporary implants and for controlled local delivery systems of therapeutic ions/molecules to targeted sites. In particular, the incorporation of antibacterial agents, slowly released as the implant degrades, can prevent biomaterial-related infections, *e.g.* post-surgery.

Despite several works presented on silicate-based glasses, much less work has been done on the analogous phosphate systems. In particular, mesoporous silicate-based glasses (MSG), have been widely studied, whilst mesoporous phosphate-based glasses (MPG) have been synthesized only very recently for the first time.^3^ This is because the PG network is much more prone to collapse and crystallisation than silicate networks under the heating process required to obtain the porous scaffold.^13^ MPG cannot be obtained using the traditional method of synthesis of PG, the melt-quenching (MQ) process, which requires fast solidification of powder oxides melts. MPG can instead be prepared using the sol–gel (SG) method combined with supramolecular templating. The sol–gel method is a wet chemical bottom-up technique based on the hydrolysis and polycondensation of alkoxide precursors in solution. Being an in-solution process, the SG method has several advantages over the MQ technique. The composition of the final glass can easily be controlled, and the morphology (desired bulk shape, nanofibers, nanospheres) can be tailored to the desired application. The use of surfactants (supramolecular templating), allows the production of scaffolds with high surface area and controlled/tailored porosity.^13^ At the critical micellar concentration (CMC), surfactants self-assemble into specifically shaped micelles, the shape, and size being dependent on the specific surfactant used. The surfactant is then removed *via* calcination or solvent exchange, and pores having the sizes of the micelles are left in the inorganic material. The surfactant templating method has been extensively used to produce MSG such as MCM-41 and SBA-15. However, to the knowledge of the authors, only two manuscripts have been published to date on MPG prepared using SG combined with supramolecular chemistry, both based on the system P_2_O_5_–CaO–Na_2_O, undoped^3^ and doped with 1, 3, and 5 mol% of Sr^2+^ for enhancement of bone healing.^14^

One of the significant advantages of mesoporous materials is the loading efficiency of therapeutic species, given the high porosity and surface area, and the capability of hosting the species inside the mesopores. In particular, the use of metallic ions as antibacterial species embedded in the pores of the MSG has been widely explored.^15^ However, the possibility of embedding antibacterial ions into the pores of MPG systems has not been explored to date. Antibacterial glasses can find application both in hard tissue engineering, as the bond healing process is often delayed by a bacterial infection (osteomyelitis), and in wound healing, as patients affected by chronic and acute wounds are at high risk of constant (and often life-threatening) infections. Of particular interest is the controlled, continuous delivery of antibacterial metallic ions (*e.g.* Cu^2+^, Zn^2+^, Ag^+^, Ce^3+^/Ce^4+^, and Ga^3+^) from the pores of the bioresorbable MPG directly to the damaged site.^16^ This would avoid systematic conventional antibiotic administration treatment (oral and injection) which often requires high doses; moreover, metallic ions have a low tendency to develop antimicrobial resistance (AMR).

In the present work, antibacterial Cu ions were embedded in an MPG at different loadings (1, 3, and 5 mol%); copper was chosen given that its antibacterial activity is well known and it is considered a potential alternative to conventional antibiotics due to the broad spectrum of antibacterial properties and reduced risk of AMR.^17^ Moreover, Cu^2+^ ions have been shown to have other important biomedical properties such as stimulation of collagen deposition, and enhancement of angiogenesis and osteogenesis.^18–21^ Cu-doped silicate-based glasses have been studied for their antibacterial activities and very recently, ordered Cu-doped silicate glasses have been presented as multifunctional platforms for bone tissue engineering.^22^ A limited number of studies have been presented on Cu-doped PG prepared *via* MQ including fibers active against *S. epidermidis*^23^ and bulk glasses active against *S. sanguis.*^24^ Non-porous PG systems prepared in-solution have also been recently shown active against *S. aureus.*^25^ However, no previous work has been presented on MPG prepared *via* SG embedded with antibacterial copper ions.

## 2. Materials and methods

### 2.1 Synthesis

The following chemical precursors were used without further purification; *n*-butyl phosphate (1 : 1 molar ratio of mono OP(OH)_2_(OBu) and di-butyl phosphate OP(OH)(OBu)_2_, Alfa Aesar, 98%), calcium methoxyethoxide (ABCR, 20% in methoxyethanol), sodium methoxide (NaOMe, Aldrich, 30 wt% in methanol), copper acetate (Cu-acetate, Aldrich, 98%), ethanol (EtOH, Fisher, 99%), and Pluronic (P123 – *M*_n_ = 5800 g mol^−1^, Aldrich).

**Fig. 1:**
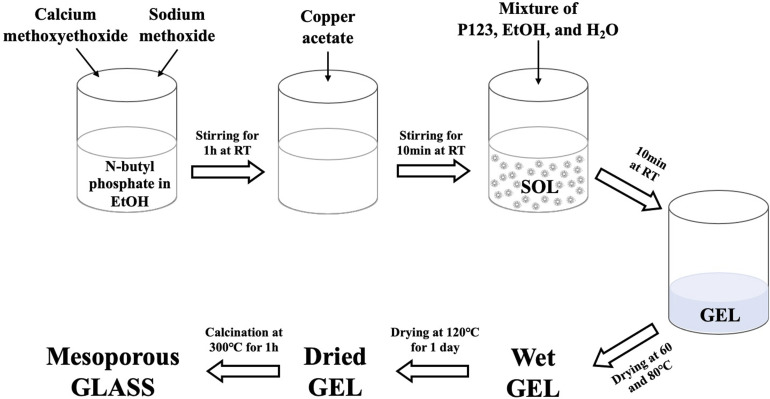
Flow diagram of the sol–gel method used for the synthesis of Cu-doped MPG.

To prepare the undoped glass, 1.7 g of *n*-butyl phosphate were added to 5 mL of EtOH in a dried vessel and left magnetically stirring for 10 min. 3.5 g of Ca-methoxyethoxide and 0.5 g of NaOMe were then added dropwise into the mixture while stirring; the solution was kept under stirring for about 1 h. The Cu-doped glasses containing 1, 3, or 5 mol% of Cu ions were prepared by adding 0.05, 0.10, and 0.15 g of Cu-acetate into the mixture, respectively; the amount of NaOMe added was reduced accordingly. The mixture was allowed to react for a further 10 min. Finally, a solution consisting of 3.0 g P123, 5 mL EtOH and 2.5 mL H_2_O was added to the mixture and allowed to react for a further 10 min. The mixtures were poured into glass containers and allowed to gel at room temperature. Gelation occurred after about 5 min; gels were then aged for 1 day at room temperature. The drying process consisted of heating the gel at 60 °C for 2 days, at 80 °C for 2 days, and finally at 120 °C for 1 day. The samples were then calcined at 300 °C at a heating rate of 1 °C min^−1^ and held at this temperature for 1 h. The obtained glasses were ground at 10 Hz (MM301 milling machine, Retsch GmbH, Hope, UK) to obtain fine powders and then sieved in the size range of 106–200 μm (Endecotts Ltd, London, UK). The sol–gel preparation of Cu-doped MPG is outlined in Fig. 1. Glasses will be hereafter indicated as MPG-und (undoped) and MPG-CuX (doped) where X = mol% Cu.

### 2.2 Structural characterization

X-ray powder diffraction (XRD, PANalytical X'Pert, Royston, UK) was performed on powdered samples in flat plate *θ*/*θ* geometry using Ni filtered Cu Kα radiation. Data were collected using a PIXcel^1D^ detector over a 2*θ* range of 10 to 90° with a step size of 0.05° and a time per step of 12 s.

Scanning Electron Microscopy (SEM) images were acquired with a JSM-7100F instrument (Jeol, Welwyn, UK) with an accelerating voltage of 10.0 kV. The samples were mounted onto aluminium stubs using carbon conductive tape. Pore sizes were measured using the Image-pro plus software (Media Cybernetics, USA). The exact composition of the glasses was evaluated using Energy Dispersive X-ray spectroscopy (EDX, MagnaRay, ThermoFisher, Hemel Hempstead, UK) performed using SEM operating at 20.0 kV.

Solid state ^31^P MAS-NMR spectra were recorded at a Bruker Avance III spectrometer, operating at a magnetic field strength of 14.1 T corresponding to a ^31^P Larmor frequency of 242.938 MHz using direct excitation with a 90° pulse and 60 s recycle delay at the ambient probe temperature (∼25 °C). Powdered samples were loaded into 4.0 mm outer diameter zirconia rotors, spun at 12 kHz, and for each sample, 16 scans were acquired. Spectra were referenced to 85% H_3_PO_4_ solution at 0 ppm. The center band resonances were fitted using the Dmfit software package.^26^

Fourier Transform Infrared (FTIR) spectroscopy was acquired using an FTIR-2000 instrument (PerkinElmer, Seer Green, UK) equipped with Timebase software with an attenuated total reflectance accessory (Golden Gate, Specac, Orpington, UK) in the range of 4000–600 cm^−1^ at room temperature.

N_2_ adsorption–desorption analysis was performed on a Gemini V instrument (Micromeritics, Hertfordshire, UK); in particular, the specific surface area was assessed by using the Brunauer–Emmet–Teller (BET) method.

Small-Angle X-ray Scattering (SAXS) measurements were conducted using the MOUSE instrument (Bundesanstalt für Materialforschung und –prüfung (BAM), Berlin, Germany).^27^ X-rays were generated from a microfocus X-ray tube, followed by multilayer optics to parallelize and monochromatize the X-ray beam to the Cu Kα wavelength (*λ* = 0.154 nm). Scattered radiation was detected on an in-vacuum Eiger 1M detector (Dectris, Switzerland), which was placed at multiple distances between 138–2507 mm from the sample. The resulting data were processed and scaled using the DAWN software package in a standardized, complete 2D correction pipeline with uncertainty propagation.^28,29^ The data were fitted and analysed using McSAS, a Monte Carlo method to extract form-free size distributions.^30^

### 2.3 Degradation study

10 mg of each sieved powdered sample were immersed in 10 mL of deionised water (Veolia Water, Elga Centra, resistivity 18.2 MΩ cm) for a period of 1, 3, 5, and 7 days. The experiments were carried out in triplicate (*n* = 3). The resulting suspensions for each time point were then centrifuged at 4800 rpm for 10 min to separate the sample particles from the solution. Phosphorus, calcium, sodium and copper in solution were subsequently measured by ICP-OES (720ES-Varian, Crawley, UK). Calibration across the predicted concentration range was performed using standard solutions (ICP multi-element standard solution, VWR). All samples and standards were diluted in a 1 : 1 ratio with 4% HNO_3_ (Honeywell, Fluka™) and a blank solution (2% HNO_3_, Trace Metal Analysis, Fisher Chemical) was used as a reference under standard operating conditions.

### 2.4 Antibacterial study

A quantitative Agar Dilution Method (ADM) was used to determine the bactericidal effects of the mesoporous phosphate-based glasses doped with 1, 3, or 5 mol% of Cu ions when in contact with the *S. aureus* NCTC 8325 and *E. coli* K12 strains for 3 days. Universal tubes with 10 mL Tryptic Soy Broth (TSB) (Oxoid, Sigma Aldrich) were inoculated with 10 mg of glass and 10 μL culture of *S. aureus* and *E. coli* at 10^6^ colony forming units per millilitre (CFU mL^−1^). Tubes were incubated at 37 °C for 3 days with a constant rotation of 250 rpm. The samples were collected every 24 h to determine bacterial viability. The experiment was conducted in three biological replicates for each sample and the undoped glass was used as a negative control. Bacterial viability is expressed as log10 CFU mL^−1^ and error bars represent standard deviations (two-way ANOVA, Dunnett's multiple comparison tests, for each time point).

## 3. Results and discussion

### 3.1 X-ray diffraction

**Fig. 2:**
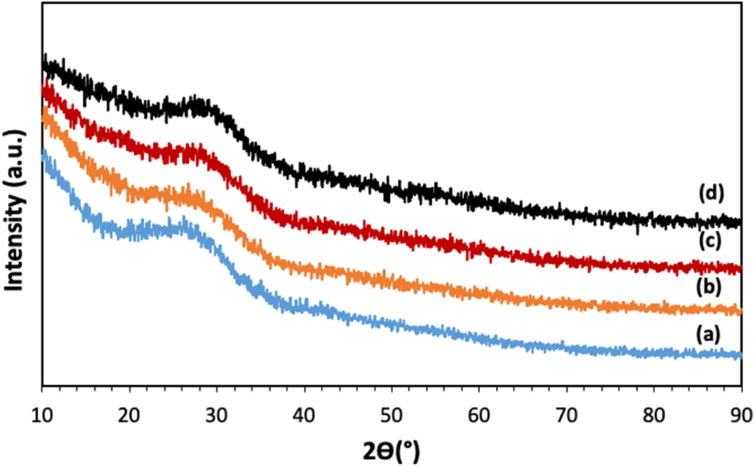
XRD patterns of (a) MPG-und, (b) MPG-Cu1, (c) MPG-Cu3, and (d) MPG-Cu5.

The XRD patterns of all samples, reported in Fig. 2, show a broad halo centered around 2*θ* ∼ 27°, with no Bragg peaks, indicating that all samples are fully amorphous and that the calcination at 300 °C and the addition of Cu ions up to 5 mol% do not induce crystallization.

### 3.2 Assessment of glass compositions

The chemical composition of all glasses was identified using SEM equipped with an EDX detector. Elemental compositions expressed in terms of oxide mol% are reported in Table 1. The compositions were chosen based on previous studies on undoped MQ and SG made PG; systems containing P_2_O_5_ and CaO in the range of 40–55 mol% and 30–40 mol% respectively, were shown to have good bioactivity and biocompatibility.^11,31^

**Table 1:** Compositions of MPG in terms of mol% of oxides

Glass code	Oxides (mol%)			
	P_2_O_5_	CaO	Na_2_O	CuO_*x*_
MPG-und	45.0 (±1.2)	36.0 (±0.9)	19.0 (±0.5)	—
MPG-Cu1	44.7 (±1.0)	36.4 (±0.7)	17.7 (±0.4)	1.2 (±0.3)
MPG-Cu3	44.3 (±1.3)	36.6 (±1.0)	16.2 (±0.6)	2.9 (±0.5)
MPG-Cu5	45.2 (±1.1)	35.4 (±0.8)	14.6 (±0.5)	4.8 (±0.5)

### 3.3 Scanning electron microscopy (SEM) analysis

**Fig. 3:**
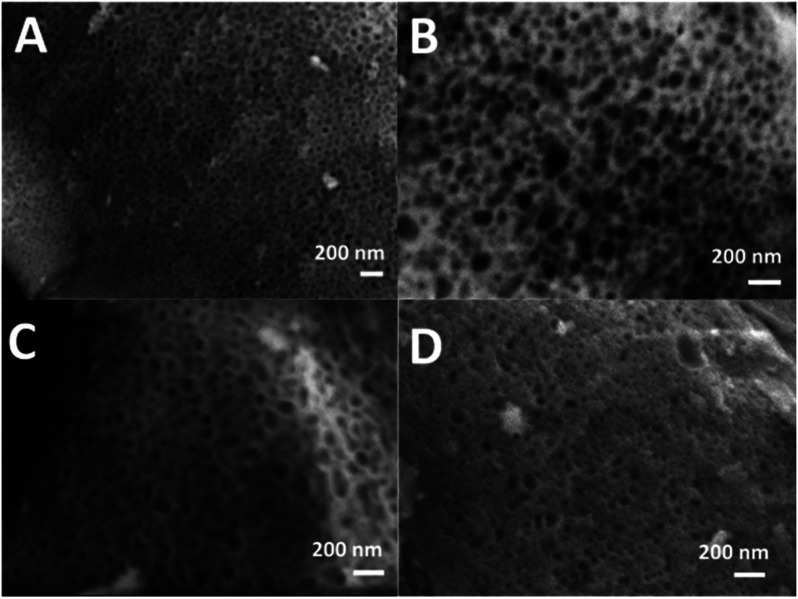
SEM images of (A) MPG-und, (B) MPG-Cu1, (C) MPG-Cu3, and (D) MPG-Cu5.

**Fig. 4:**
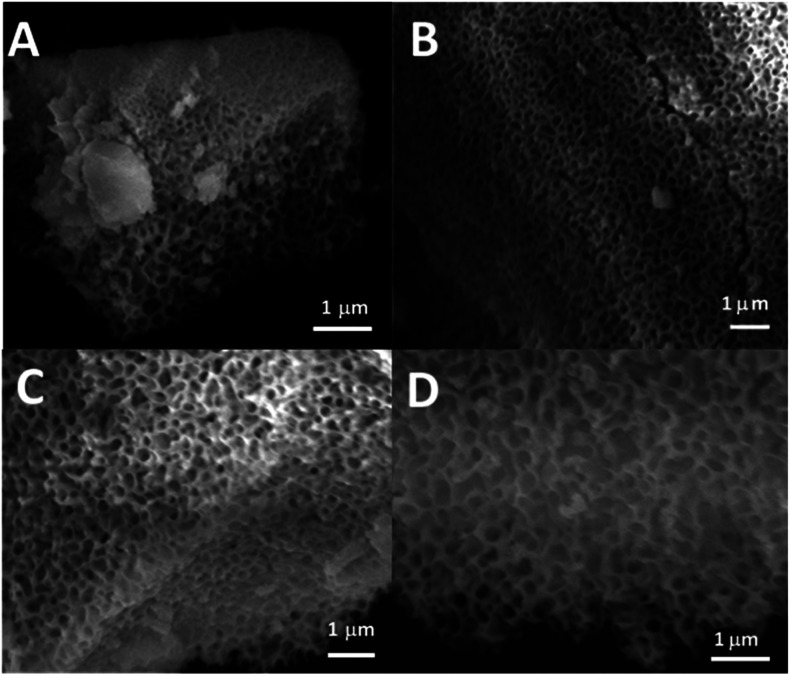
SEM images of MPG-Cu3.

SEM images of all glasses show extended porosity, generated by the removal of the micelles of the surfactant P123 by calcination (Fig. 3A–D). Pores sizes are in the range of 8–20 nm with walls about 4–5 nm thick. This is in agreement with typical values found in MPG prepared using P123 as a surfactant.^32^ These results confirm that MPG can be obtained similarly to MSG using P123. However, the SEM analysis also shows the presence of pores bigger than 20 nm, with sizes extending to macropores (up to 200 nm). Representative images of the sample MPG-Cu3 show the presence of pores in the range of 50, 45, 82, and 140 nm (Fig. 4A, B, C and D, respectively). These bigger pores could have been produced by the merging of smaller mesopores during the calcination process, as suggested by the worm-like structure, particularly evident in Fig. 4C and D.

### 3.4 Small-angle X-ray scattering (SAXS) analysis

**Fig. 5:**
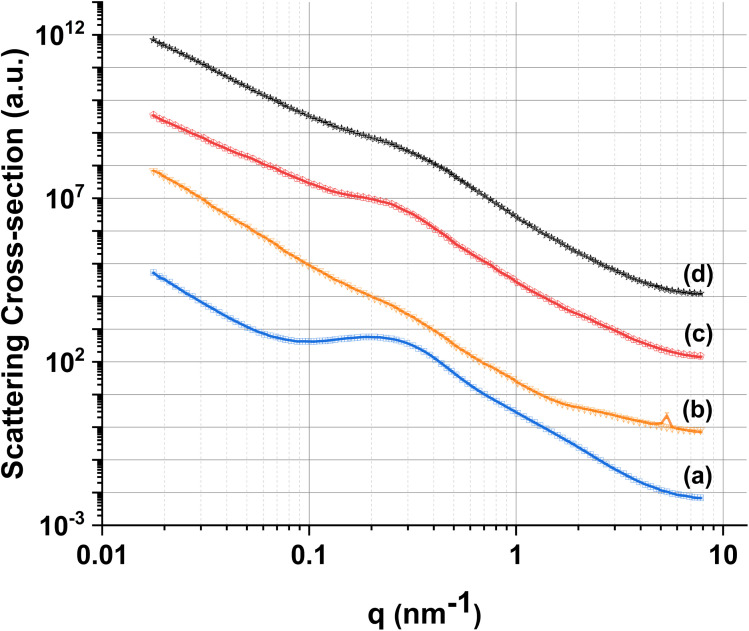
Experimental SAXS data (lines) and fits (symbols) for (a) MPG-und, (b) MPG-Cu1, (c) MPG-Cu3, and (d) MPG-Cu5.

In order to further investigate the pore structure of MPG, SAXS data were collected (Fig. 5). SAXS is one of the few experimental techniques that can be used to study the structure of porous materials in the full range from 1 to around 400 nm, which makes it particularly suitable for the study of mesoporous materials.^33^ All samples show the presence of two distinct pore systems, with populations centered around 1.5 and 11 nm (Table 2). An upturn in the scattering signal is also observed towards low-q, indicating the presence of larger structures (*e.g.* from larger pores), in agreement with SEM images.

**Table 2:** Mean radii of pore populations from MPG glasses derived from fits of SAXS data

Size distribution range (nm)	MPG-und	MPG-Cu1	MPG-Cu3	MPG-Cu5
0.6–4	2.01 ± 0.14	1.19 ± 0.06	1.54 ± 0.07	1.89 ± 0.14
4–40	11.07 ± 0.04	16.25 ± 0.28	14.04 ± 0.10	13.69 ± 0.14
40–200	141.6 ± 2.51	133.9 ± 0.94	109.6 ± 2.21	122.9 ± 2.02

### 3.5 Adsorption–desorption of N_2_ at 77 K

**Fig. 6:**
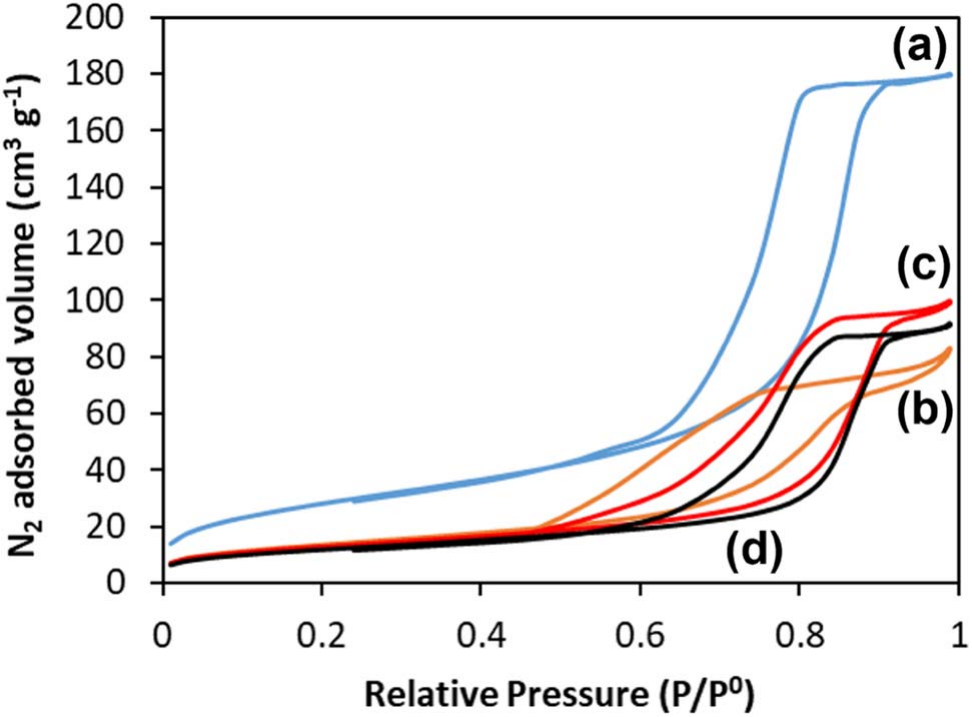
N_2_ adsorption and desorption isotherms at 77 K of (a) MPG-und, (b) MPG-Cu1, (c) MPG-Cu3, and (d) MPG-Cu5.

One of the great advantages of highly porous materials is the increased surface area compared to bulk systems. The surface areas of all glasses were measured using the adsorption–desorption of N_2_ at 77 K; information on pore size, volume, and morphology were also obtained. The N_2_ adsorption–desorption isotherms of all the glasses are shown in Fig. 6. The presence in all samples of isotherms with a hysteresis loop starting at *P*/*P*_0_ ∼ 0.45 confirms the presence of mesopores. These isotherms, classified as type IV, arise from capillary condensation and evaporation in mesoporous materials. The shape of the hysteresis loop is type H1 for MPG-und, which is indicative of a hexagonal arrangement of cylindrical pores open at both ends. The surfactant used (P123) is known to form cylindrical micelles, which aggregate forming two dimensional hexagonal arrangements. This explains the presence of hexagonal arrays of unidirectional cylindrical pores after the removal of the micelles by calcination. The shape of the hysteresis for the doped samples is slightly distorted, but compatible with the presence of mesopores.

The surface areas calculated using the BET model, pore sizes and pore volumes are reported in Table 3. The MPG-und sample has the highest surface area (124 m^2^ g^−1^) and an average pore size of around 12 nm. As the copper content increases, the surface area decreases, reaching the lowest value of 67 m^2^ g^−1^ for the MPG-Cu5 sample. However, pore size does not change significantly with the copper content, in agreement with previously investigated Sr^2+^ doped MPG.^14^

**Table 3:** Textural properties of MPG glasses derived from nitrogen adsorption/desorption isotherms

Sample code	Surface area (m^2^ g^−1^)	Pore size (nm)
MPG-und	124	11.8
MPG-Cu1	87	12.6
MPG-Cu3	73	12.0
MPG-Cu5	67	12.2

### 3.6 ^31^P MAS NMR

**Fig. 7:**
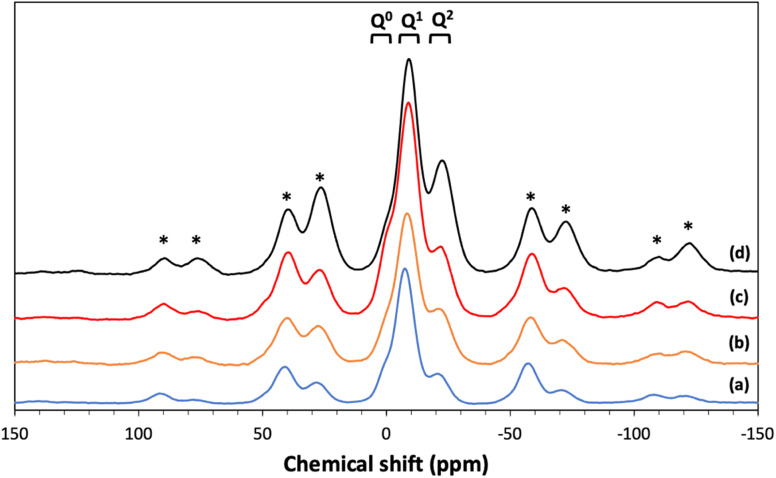
^31^P MAS NMR spectra of (a) MPG-und, (b) MPG-Cu1, (c) MPG-Cu3, and (d) MPG-Cu5. * = spinning sidebands.

^31^P MAS NMR was used to characterize the phosphate network structure, the connectivity of the phosphate units and the local environment around each phosphorus species. All spectra are presented in Fig. 7 and the corresponding spectral parameters are reported in Table 4. Resonances are assigned using the Q^*n*^ notation, where *n* represents the number of bridging oxygens between phosphates units. Three main resonances are observed corresponding to Q^0^ groups in the range of 0.11 to 1.33 ppm, Q^1^ groups in the range of −7.4/−9.0 ppm and Q^2^ groups in the range of −21.1/−22.6 ppm.^25^

**Table 4:** ^31^P MAS-NMR chemical shifts (*δ*_iso_) and relative intensities (*I*%) for the studied glass compositions

Composition	Q^*n*^	*δ*_iso_	%Int
MPG-Und	Q^0^	1.33	5.5
	Q^1^	−7.44	70.0
	Q^2^	−21.09	24.5
MPG-Cu1	Q^0^	0.11	5.1
	Q^1^	−9.04	51.5
	Q^2^	−22.59	43.5
MPG-Cu3	Q^0^	0.22	8.5
	Q^1^	−8.91	67.6
	Q^2^	−22.28	23.9
MPG-Cu5	Q^0^	0.84	6.0
	Q^1^	−8.38	61.9
	Q^2^	−21.98	32.2

### 3.7 Fourier transform infrared spectroscopy (FT-IR) analysis

**Fig. 8:**
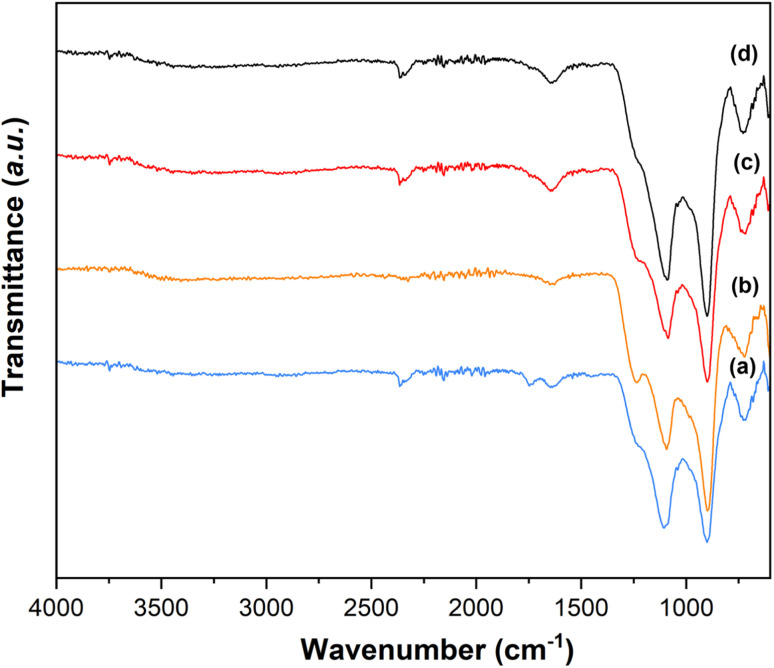
FTIR spectra of (a) MPG-und, (b) MPG-Cu1, (c) MPG-Cu3, and (d) MPG-Cu5.

The presence of Q^1^ and Q^2^ units in the phosphate chains was confirmed by the FTIR measurements. FTIR spectra of all samples are reported in Fig. 8.^25,34^ The peak at 730 cm^−1^ can be assigned to the symmetrical stretching *υ*_s_ (P–O–P) mode, while the peak at 900 cm^−1^ can be assigned to the asymmetrical stretching *υ*_as_ (P–O–P) mode (Q^2^ phosphate units). The peaks at 1100 cm^−1^ and 1235 cm^−1^ are assigned to asymmetrical *υ*_as_ (PO_3_)^2-^ and *υ*_as_ (PO_2_) modes that can be related to Q^1^ and Q^2^ phosphate units, respectively.

### 3.8 Dissolution study

**Fig. 9:**
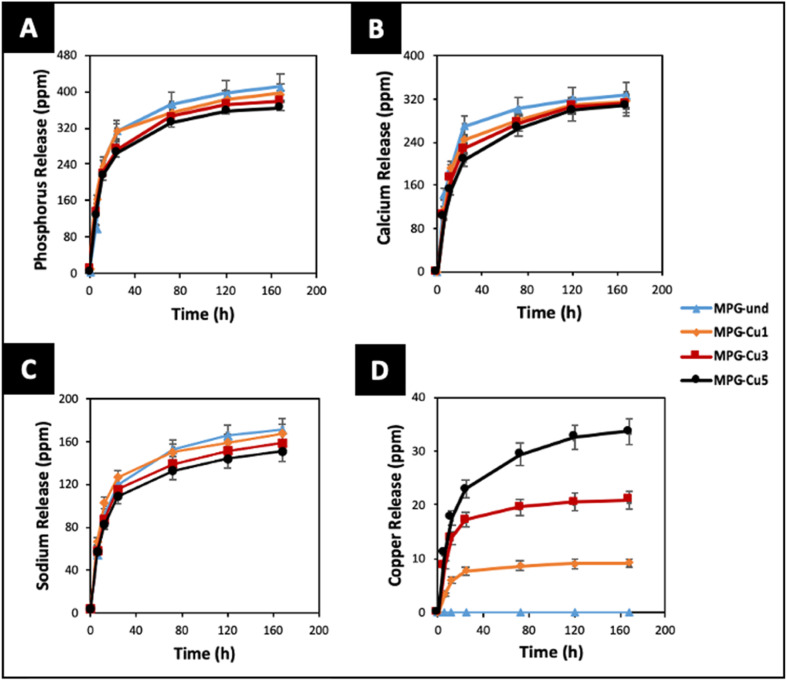
Release of (A) phosphate (ppm P), (B) calcium, (C) sodium and (D) copper ions in deionised water up to 7 days by ICP-OES. Error bars are standard deviation (*n* = 3).

One of the main applications of MPG is the controlled delivery of therapeutical species. Therefore, the knowledge of dissolution rates and ion release over time are of paramount importance. The degradation of all glasses in deionized water was studied for 7 days. The release profiles of P, Ca^2+^, Na^+^, and Cu ions, identified *via* ICP-OES, are reported in Fig. 9. It has to be noted that ICP-OES measures the release of P; however, phosphate anions are the actual species released by the MPG. The highest release for all species occurs in the first 24 hours. Phosphate, Ca and Na release profiles do not change significantly with copper loading in the first 24 h. However, clear differences can be seen in the release profiles over the following 6 days. In particular, the MPG-und glasses released the highest amount of phosphate, Ca and Na; the amount of phosphate, Ca and Na released decreased with increasing copper content. As expected, release of copper is dependent on its content, clearly increasing with the copper loading. In contrast to phosphate and Na release, dose dependent release of copper is also seen in the first 24 h. The decrease in phosphate release could be related to the cross-linking effect of Cu ions that strengthen the connection between phosphate chains, increasing their strength in solution.

### 3.9 Antimicrobial study

**Fig. 10:**
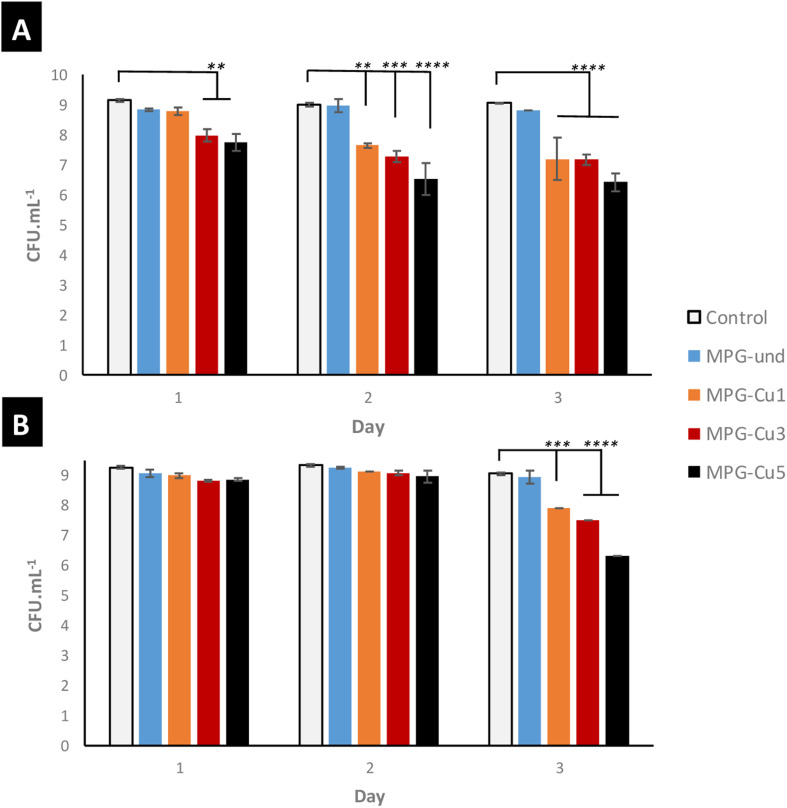
Antibacterial activity of all MPG doped with 1, 3, and 5 mol% of Cu against *S. aureus* NCTC 8325 (A) and *E. coli* K12 (B). ** = *p* ≤ 0.01, *** = *p* ≤ 0.001 **** = *p* < 0.0001.

The antibacterial activity of all glasses was measured against *S. aureus* and *E. coli* bacterial strains (Fig. 10) for 3 days. All the MPG-Cu samples show a progressive reduction in the log 10 of the mean number of viable counts from day 1 to 3 against both bacterial strains. As expected, the antibacterial activity increases with increasing mol% of Cu; in particular, MPG-Cu5 was the most effective sample for both bacterial strains with statistically significant reduction up to 3 log difference (*p* < 0.0001). MPG-Cu3 and MPG-Cu5 show activity against *S. aureus* from day 1. At days 2 and 3, the activity is also evident for MGP-Cu1. A similar trend was observed against *E. coli*. However, MPG-Cu seems to be more effective in reducing *S. aureus* than *E. coli*. It is interesting to observe that the undoped samples show a minor antibacterial activity that does not change over time. Similar observations have been reported for PG prepared by MQ,^35^ SG^36^ and PG prepare *via* SG doped with Sr^2+^.^14^ This could be due to a slight change in pH during glass degradation.

## 4. Discussion

The porous structure of MPG has been investigated using three complementary techniques: SEM imaging, SAXS, and absorption–desorption analysis at 77 K. Direct imaging *via* SEM and SAXS analysis shows consistent results, indicating the presence of mesopores around 2–20 nm in size and macropores > 100 nm. The simultaneous presence of macropores and mesopores (hierarchical porosity) has been considered beneficial for tissue engineering applications.^37^ Due to the nature of the technique, absorption–desorption analysis at 77 K cannot detect macropores. However, the presence of mesopores is confirmed by absorption–desorption analysis that reveals an average pore size of 12 nm. Doping of the glasses up to 5 mol% Cu does not seem to affect the size of the mesopores; however, the surface area is affected, being almost halved for MPG-Cu5 (67 m^2^ g^−1^) compared to the undoped glass (124 m^2^ g^−1^). Even if the surface areas are lower than typical mesoporous silicate-based glasses, this is a remarkable result given that the calcium phosphate-based glasses are known to have a much weaker network structure than that of silicate-based systems.

**Fig. 11:**
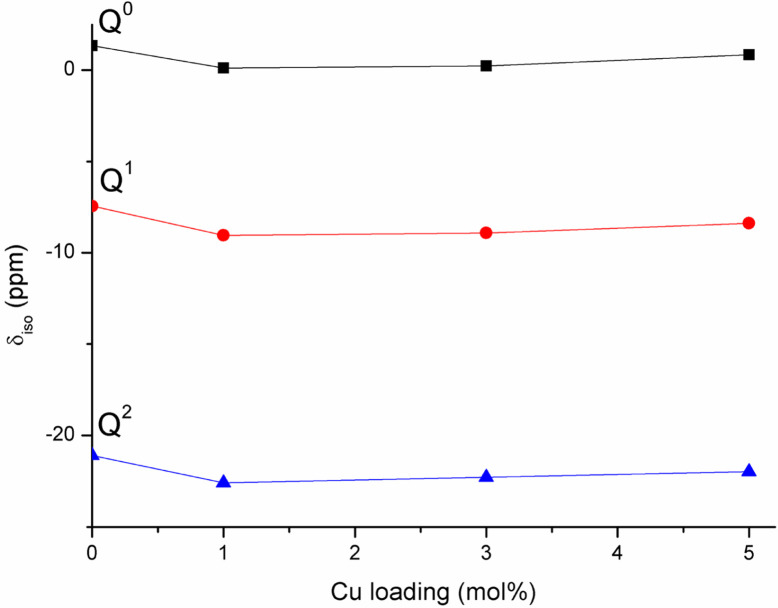
Variation of ^31^P isotropic chemical shifts of Q^*n*^ species with Cu loading in the studied MPG.

The ^31^P MAS NMR spectra show that Q^2^, Q^1^ and Q^0^ phosphate species are present, with Q^1^ being the dominant species in all glass compositions. This is significantly different from the structure of undoped PG of similar composition (45P_2_O_5_ : 30CaO : 25Na_2_O) prepared *via* MQ^38^ and *via* untemplated SG, that have Q^2^ as the predominant phosphate species (∼77% Q^2^ and 23% Q^1^ in both systems). The results are however consistent with other MPG systems prepared *via* SG doped with Sr^2+^ ions^14^ and PG doped with Cu prepared using an in solution-single step technique.^25^ The predominance of Q^1^ species in the MPG glasses suggests a lower degree of network condensation (*i.e.* higher O : P ratio) than would be expected from the starting composition. The explanation for this lies in the use of P123 as a templating agent in the synthesis of MPG glasses. Hydrolysis leads to the formation of P–O–C linkages leading to a breaking up of the phosphate network and a raising of the O : P ratio, without the need for additional modifying cations. Indeed, the absence of O–H vibrations in the FTIR data appears to support the elimination of water. Fig. 11 shows the compositional variation of isotropic chemical shift of the three Q^*n*^ species in the present system. It is evident that for all species there is a significant upfield shift of the signal (more negative values) on initial Cu substitution, but that as the Cu content increases in the Cu containing glasses the signal shifts downfield (more positive values). This suggests that for the Cu containing compositions, there is an initial increase in shielding with the addition of Cu followed by an decrease in shielding with further addition of Cu. This trend is different from that seen in 40P_2_O_5_ : (60 − *x*)Na_2_O : *x*CuO glasses where an increase in shielding with increasing Cu content was observed over a wide compositional range (10 ≤ *x* ≤ 40).^39^

Cu has been found to adopt both Cu^+^ and Cu^2+^ oxidation states when incorporated into phosphate glasses.^40^ In the present system, it might be speculated that at least some of the copper enters the glass in a monovalent state *i.e.*, a single Cu^+^ cation replaces two Na^+^ ions. This would have the effect of lowering the overall cation charge in the system resulting in an increase in shielding. Additionally, lowering the overall cation charge would lower the O : P ratio resulting in a greater degree of condensation *i.e.*, more Q^2^ species, consistent with the observed data in Table 4. Furthermore, the 3d^9^ electron configuration of Cu^2+^ would be expected to be paramagnetic, whereas the 3d^10^ configuration of Cu^+^ is diamagnetic. The appearance of the ^31^P spectra of the Cu containing glasses in Fig. 7 differs little from that for the MPG-und glass with no evidence of paramagnetic broadening nor of additional paramagnetic sidebands and thus is consistent with Cu being in the lower oxidation state. The decrease in shielding on increasing the Cu content in the MPG-Cu3 and MPG-Cu5 glasses suggests a change in the role of Cu. Copper is a network intermediate and has been found to adopt both network forming and modifying roles in phosphate glasses depending not only on Cu concentration but also on O : P ratio.^39^ A network forming role for Cu would involve the formation of covalent P–O–Cu linkages, resulting in an increase in shielding at the ^31^P nucleus compared to the more ionic P–O^−^⋯Cu^*n*+^ interaction when Cu is in the modifying role. The results suggest that Cu initially enters the network in a network modifying role but that as the copper content increases it switches to a more network forming role.

Dissolution studies have shown that phosphate, Ca^2+^ and Na^+^ ions can be released in a controlled way. In particular, the release of phosphate, Ca, and Na followed a similar pattern, with very similar dissolution occurring in the first 24 h for the different compositions, followed by a progressive decrease of phosphate, Ca, and Na release with increasing Cu content over 7 days. This suggests that Cu ions might act as a cross-linker between the phosphate chains, slowing down their dissolution. Therefore, the degradation of the glasses can be tailored by controlling the loading of Cu as a dopant. The increase of Cu ions released with increasing Cu content is in agreement with results reported on PG fibres prepared *via* MQ^23^ and with PG prepared *via* in solution-single step^25^ and coacervation techniques.^41^ The same trend has been reported upon the release of Zn^2+^ and Sr^2+^ from MQ PG and Sr^2+^ from SG MPG.^42,43^

The capability of releasing Cu ions has been linked to the antibacterial activity which increases with copper content. The glasses that contain and release the highest amount of Cu ions (MPG-Cu3 and MPG-Cu5) were shown to be the most effective in killing the bacteria *S. aureus* from day 1 by significantly reducing the number of viable bacteria (2 and 3 log reduction for MPG-Cu3 and MPG-Cu5, respectively), whereas MPG-Cu1 starts to be active only from day 2. Against *E. coli*, the antibacterial activity is evident only after day 2 for all compositions, with a clear dependence on Cu content at day 3. The higher resistance of *E. coli* to the antibacterial action of Cu ions compared to *S. aureus*, could be explained by the difference in the structure of the cell membrane of the two bacteria. It has been shown that the mechanism of action of Cu ions involves damaging the membrane and infiltration of the cell; Gram-negative bacteria such *E. coli*, have an additional outer membrane compared to Gram-positive bacteria such *S. aureus* which makes them less susceptible to antibacterial agents.^44^

## 5. Conclusions

This study presents the first example of antibacterial mesoporous phosphate-based glasses in the P_2_O_5_–CaO–Na_2_O–CuO system (CuO_*x*_ = 1, 3, or 5 mol%) prepared *via* the in-solution method of sol–gel combined with supramolecular templating. SEM images showed that all prepared samples have an extended porous structure, with mesopores in the range 8–20 nm but also the presence of bigger pores up to 200 nm. These results were confirmed by SAXS analysis that shows pores in the range of 2–20 nm and some macropores of size >100 nm. The presence of mesopores was confirmed by N_2_ adsorption–desorption analysis at 77 K that showed a porosity typical of mesoporous materials with a hexagonal arrays of pores. XRD, FT-IR, and ^31^P MAS-NMR results revealed that all samples are amorphous and consist of mainly Q^1^ phosphate units. The role of Cu in the MPG appears to change from network modifying to network forming with increasing Cu content. Additionally, the lack of evidence for paramagnetism suggests Cu may well be in the monovalent state. The surface area, evaluated *via* SEM images and N_2_ adsorption–desorption analysis, shows a slight decrease upon the addition of Cu ions (from 123 to 67 m^2^ g^−1^) with an average pore size of 12 nm. A study of degradation suggested that as the Cu content increases, the glass degradation rate decreases. Measurement of antibacterial activity shows that there is a direct correlation between antibacterial activity and Cu content, with MPG-Cu5 being the most active against Gram-positive and Gram-negative bacteria with statistically significant bacteria reduction. The prepared mesoporous Cu-doped phosphate-based glasses have great potential tissue engineering applications as materials for the controlled delivery of antibacterial Cu ions in a controlled manner into the implanted site.

## Conflicts of interest

There are no conflicts of declare.

## Supplementary Material
